# The timescales of global surface-ocean connectivity

**DOI:** 10.1038/ncomms11239

**Published:** 2016-04-19

**Authors:** Bror F. Jönsson, James R. Watson

**Affiliations:** 1 Department of Geosciences, Princeton University, Princeton, New Jersey 08544, USA; 2 College of Earth, Ocean and Atmospheric Sciences, Oregon State University, Corvallis, Oregon 97331-5503, USA; 3 The Stockholm Resilience Centre, Stockholm University, 118 14 Stockholm, Sweden

## Abstract

Planktonic communities are shaped through a balance of local evolutionary adaptation and ecological succession driven in large part by migration. The timescales over which these processes operate are still largely unresolved. Here we use Lagrangian particle tracking and network theory to quantify the timescale over which surface currents connect different regions of the global ocean. We find that the fastest path between two patches—each randomly located anywhere in the surface ocean—is, on average, less than a decade. These results suggest that marine planktonic communities may keep pace with climate change—increasing temperatures, ocean acidification and changes in stratification over decadal timescales—through the advection of resilient types.

## Abstract (web summary)

The adaptive capabilities of planktonic communities to climate change remain uncertain. Here, using Lagrangian particle tracking and network theory, the authors show that surface ocean currents can navigate the globe within 10 years, suggesting that marine plankton may keep pace with climate change.

## Graphical abstract

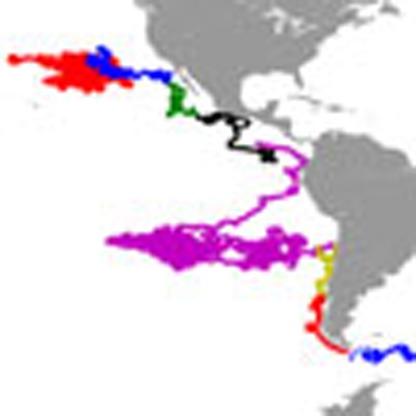

Two different paradigms are used to explain the structuring of planktonic communities (bacteria, phytoplankton and zooplankton) in ocean ecosystems. One fundamental idea is that ‘everything is everywhere but the environment selects'^1^,^2^. That is, different regions of the ocean are connected by ocean currents, resulting in potentially panmictic planktonic communities^3^. It is then the differential response of species to environmental conditions that leads to community structure^4^,^5^. The alternative is that regions of the oceans are not so well connected, and that this isolation leads to divergent evolution and hence differences in which species are where^6^,^7^. Recent work has shown that neither concept is wholly accurate, with a number of examples showing slight spatial structure on global and regional scales in marine microbial communities^8^,^9^,^10^ and sometimes strong genetic differentiation at small spatial scales^11^. Such examples suggest that dispersal limitation is important in specific areas of the ocean. However, most studies have focused on patterns of community composition, or genetic differences of individuals within a species. The key mechanism itself—dispersal by surface ocean currents—has rarely^12^ been explored and quantified.

One common method for investigating the timescales of dispersal for marine organisms is to calculate oceanographic distances by tracking virtual particles in modeled velocity fields^13^,^14^. Particle tracking has been used in a variety of ways to explain Lagrangian processes in the ocean, such as biological dispersal on regional scales^14^,^15^,^16^,^17^, connectivity between different coastal habitats like coral reefs^18^,^19^ and deep-water transport pathways^20^.

Normally, oceanographical distances are defined as the expected connectivity times, or the mean time it takes for particles to travel from one location to another^15^,^21^. Another option is to instead use minimum connectivity times (Min-T), or distances based on the fastest times that particles can travel from one location to another. Minimum connection times have major advantages over expected connection times for problems concerning global plankton communities. First, the minimum connection time is a more appropriate metric for phytoplankton and bacterial connectivity since asexually reproducing organisms have high reproductive output that attenuates low dispersal probabilities, and only a few individuals are required to ‘connect' two places, especially in terms of population genetics^22^. It is therefore possible for such organisms to exploit dispersal routes where the probability to reach a given destination is very low. Previous empirical work have also shown that minimum connection times provide better correspondence with the genetic similarity of groups^13^ and that minimum connection times are more relevant to community similarity than the mean. Second, mean or median transit times in the global ocean are not well defined, as water can recirculate eternally and, hence, every particle seeded in a given patch eventually will reach all other patches if enough time is provided. Constraining the particles to a maximum advection time or using a lower percentile of connections (for example, the fastest 20%) would create results that mainly depend on on those arbitrary cutoffs. It is challenging to identify one physically motivated timescale (loop time of the the subtropical gyres, typical time spent in the gulf stream, time to circumfer the Antarctic Circumpolar Current and so on) that is generally applicable to all regions in the ocean. The estimates of minimum connection times for the global surface ocean are stored in the form of a matrix (the Min-T connectivity matrix) where each *i*, *j* element represents the shortest transit time between a given source patch *i* and destination patch *j*.

Connectivity matrices produced from Lagrangian particle tracking tend to be highly sparse with most pairs of patches unconnected. Indeed, to estimate connection times between all pairs of patches globally, an infeasible number of Lagrangian particles^23^ would be needed. To circumvent this obstacle, a shortest path algorithm can be used to calculate missing connections. Here the network is the global ocean, with patches in the ocean as nodes and minimum connection times as edges connecting the nodes. Applied to this network, shortest path algorithms identify the shortest path between every global ocean patch pair, accounting for all possible multistep connections. For example, if there is no direct connection between nodes *A* and *D*, then these algorithms identify the multistep connection from *A*→*B*→*C*→*D*. We use Dijkstra's algorithm^24^, which is one of the most commonly used shortest path algorithms, and which fits our specific application.

Each step along these minimum-time routes may be unlikely, making the conditional multistep probability (of going from A to B to C to D...) very low as well. However, one can assume that the effect of these low probabilities is attenuated by the large reproductive output of microorganisms drifting with ocean currents. Over monthly to annual timescales, microorganisms moving with water masses can grow by the million^25^. Hence, there will still be planktonic organisms traveling along the potentially low probability paths identified here. Indeed, if one considers the dispersal of genetic material, then there need only be a small number of individuals traveling along these Min-T routes to make them evolutionarily relevant^17^,^22^,^25^.

The outcome of our study when applying Dijkstra's algorithm to a raw Min-T matrix is a full matrix that contains estimates of minimum connection times between every region of the world's surface ocean. Both the raw and full Min-T matrices are rich with spatial information, but most importantly are the distribution of minimum connection times themselves. We find that different regions in the global surface ocean are connected on very short timescales, within ∼10 years. This is in contrast to deep-water circulation, where water is thought to recirculate around the globe in roughly 1,000 years. These short surface-connection times are relevant to anyone studying dispersion in the surface ocean beyond planktonic species, including radioactive materials, plastics and other forms of pollution.

## Results

### Particle advection

We seeded particles in near-surface velocity fields from the ECCO2 1/4° × 1/4° state estimate^26^ over 9 years and advected them for 100 years by looping fields for the years 2000–2010. The resulting paths were used to estimate the shortest time taken for water to travel from one patch in the surface ocean to another. Minimum connectivity times were then calculated by aggregating the ECCO2 grid cells (Supplementary Fig. 1) into 8 × 8 patches, each approximately 2° × 2° in size (11,116 patches in total: Supplementary Fig. 2). On average, particles seeded in any given source patch reached 1,150 destination patches after 100 years of advection by ocean currents.

### Connectivity matrices

**Figure 1:**
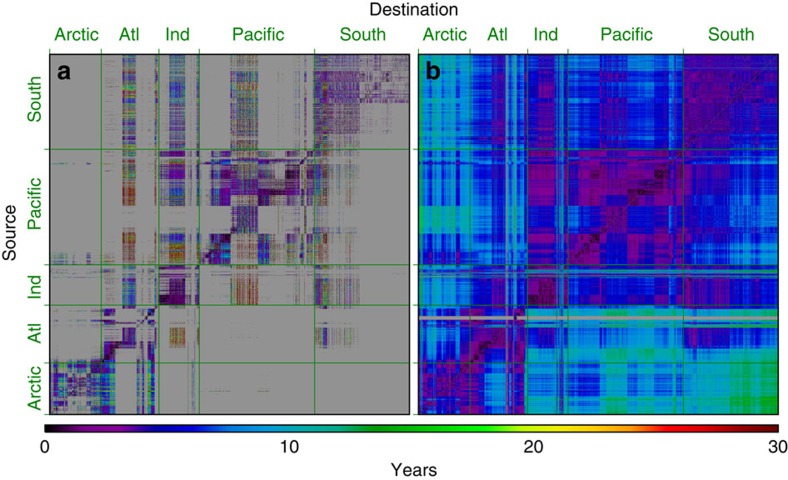
Connectivity matrices. The raw minimum time connectivity matrix (**a**) and after Dijkstra's algorithm was applied (**b**). Major oceans are delimited by green lines. The number of patches (and hence the number of rows and columns) is 11,116.

The raw Min-T connectivity matrix, produced from the 2D Lagrangian particle simulations, is highly sparse (Fig. 1a, grey areas). Connections are made primarily within each ocean basin, reflecting the computational limits of the simulation integration period (see Methods), with values hugging the diagonal. Some cross-basin connections are made, and these typically take much longer, on the order of 20–30 years. In contrast, the Min-T matrix, modified by applying Dijkstra's algorithm (Fig. 1b), is full with minimum connection times for every ocean–patch pair. Short values still hug the diagonal, but now the cross-basin connection times are shorter, on the order of 10–20 years. For example, the largest connection time values in the full Min-T matrix occur between the Arctic and Southern Oceans. Asymmetry is present, too, revealing that there are differences in the time taken to go to, and come from, two places.

### Spatial properties of connectivity

**Figure 2:**
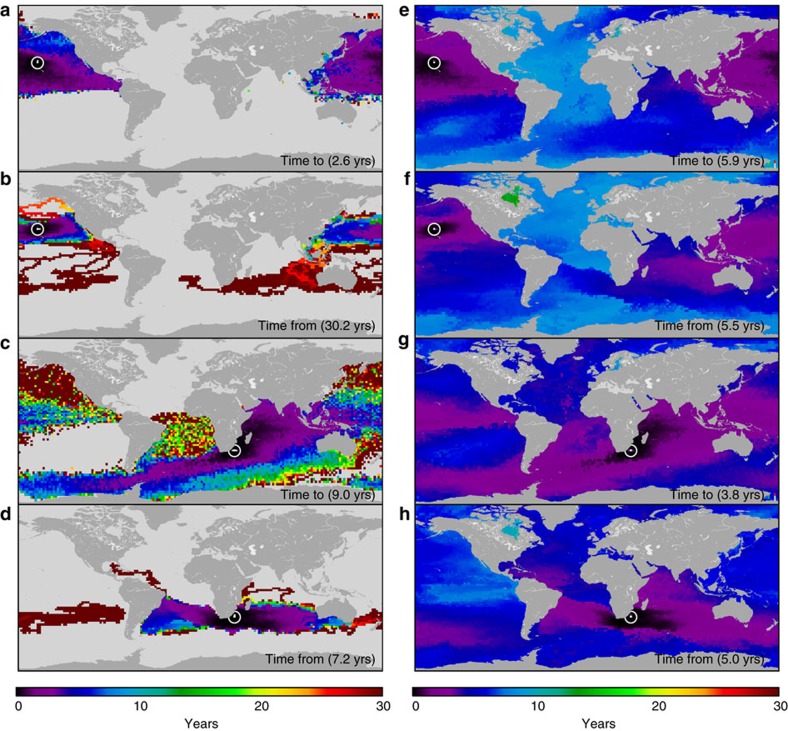
Connectivity examples. Examples of minimum connection times (Min-T) to and from two locations identified by white circle-dots: off Hawaii (**a**,**b**,**e**,**f**) and off South Africa (**c**,**d**,**g**,**h**). Times ‘to' are the shortest times taken for water from other patches to arrive at these locations. Times ‘from' are the shortest times taken for water from these locations to go to all others. The left column shows raw minimum connection times, with the large number of no-connections noted in grey, and median Min-T in parentheses. The right column panels show Min-T values generated using Dijkstra's algorithm. Here connections occur between all areas of the ocean and median values are much lower on average than those of the raw minimum connection times.

Rows of the raw and full Min-T matrices describe the minimum connection times from particular patches to all other patches in the global surface ocean. Similarly, the columns of the Min-T matrices describe the minimum time it takes for water to go from all patches to a given patch. This information is shown in Fig. 2 for two locations: Hawaii and a coastal location off of South Africa. From the raw Min-T information (Fig. 2a), the limitations of the particle tracking are evident in the large number of locations that are not connected by any particle trajectories (Fig. 2, ocean areas in grey). Of those that are connected, patches near the release point have low Min-T values relative to those locations farther away, with median connection times varying from location to location. In contrast, the full Min-T values (Fig. 2b) have connections everywhere, as expected from using Dijkstra's algorithm. Spatial structure is still seen, with some places more connected than others, but long connection times are now absent, and all median values have changed relative to their raw Min-T counterparts (Fig. 2, values in parentheses).

### Timescales of global surface ocean connectivity

**Figure 3:**
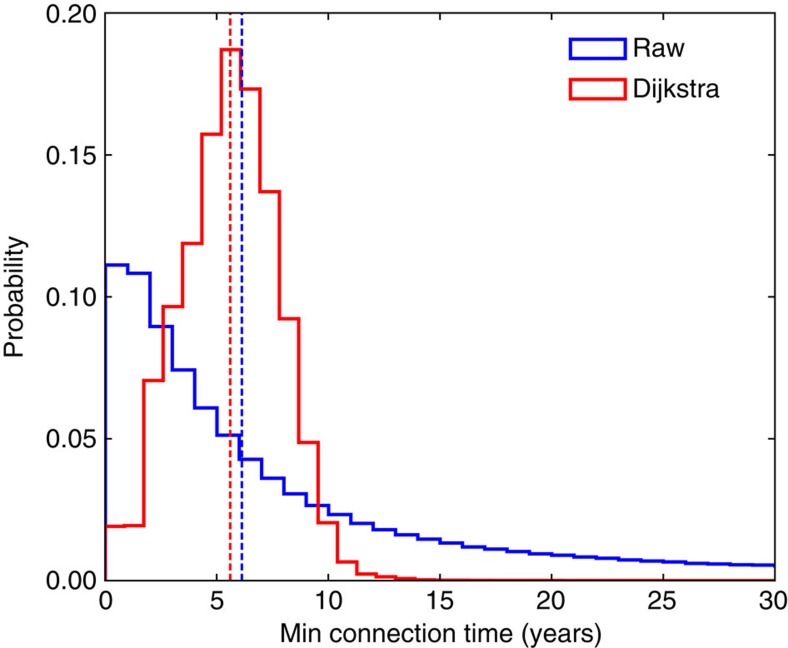
Global connectivity distributions. Probability distributions of raw minimum connections times (blue) and those produced from Dijkstra's algorithm (red). Median minimum connection times (identified by the dashed vertical lines) are 6.13 years for the raw matrix, and 6.11 years for the modified. Note that connection times shorter than 1 year for Dijkstra are in fact raw connection times.

The most notable result from our analysis is the distribution of Min-T values themselves. The distribution of raw Min-T values (Fig. 3, blue distribution) is roughly log-normal, with a median value of 6.13 years, and a long tail extending towards 100 years when the simulations were stopped. After being modified by Dijkstra's algorithm, the global distribution of minimum connection times is changed (Fig. 3, red distribution) with a median minimum connection time of 5.61 years and with the bulk of the distribution now below 15 years, showing that the global ocean can be connected over timescales of a decade. The maximum full Min-T value is still about 100 years, relating to water traveling from the Weddell Sea to the California coast. In scaling up, the average Min-T values between different ocean basins are shown in Table 1. These aggregated metrics again highlight the short connection times between ocean regions, but also show physical consistency (that is, on average basins farther away take longer time to reach).

## Discussion

Our results mirror the data from unintended, often tragic, natural experiments that quantify analogous connectivity in the Pacific Ocean. Large quantities of shoes^27^ and toys^28^ washed overboard from container ships en route from Asia to North America have been useful in estimating connectivity times by acting as drifters. Such results show timescales that are similar to, or often shorter than, our findings in the North Pacific. These kinds of drifters are, however, susceptible to wind-drift and could record faster times than our models. A more comparable experiment is the 2011 Fukushima disaster, in which a Japanese nuclear reactor released a large quantity of radioactive isotopes into the Pacific Ocean. Traces of radioactivity were detected on the Pacific Coast of the U.S. in November of 2014—3.6 years later (Ken Buessler WHOI, personal communication). Our estimated minimum connectivity time between the Fukushima release site and its detection site of the U.S. west coast is 3.5 years.

In summary, our results provide evidence for a highly connected global surface ocean with all regions connected to each other over decadal timescales. This suggests that plankton communities may keep pace with climate change through the immigration of new types that are better suited in changing local conditions^5^,^29^,^30^. Beyond this result, the utility of calculating global surface connectivity extends to its spatial information. For example, in many regional studies it is common to identify connectivity modules or subpopulations^31^ and also the location of key stepping-stone patches, which are central to maintaining the overall connectance of the system^32^,^33^. These network theoretic analyses have an applied nature, such as in the design of spatial management units^34^; but they are also important for basic research, for example in generating hypotheses about genetic or taxonomic similarity across the ocean^7^, or when testing models of community assembly^35^. Finally, it is important to note that we have only estimated the timescales of physical connectivity without addressing environmental factors such as nutrient availability or temperature gradients^36^. Gauging the effect of environmental barriers on global-scale dispersal will further contribute to our understanding of how marine communities adapt to their changing ocean environment.

## Methods

### Lagrangian particle tracking

Two-dimensional Lagrangian particle tracking was used to make our connectivity calculations^15^,^21^,^33^,^37^. We used velocity fields from ECCO2 (http://ecco2.org), a high-resolution (1/4) global ocean model that assimilates available satellite and the *in situ* data^26^, to advect particles in the surface ocean (Supplementary Fig. 1). ECCO2 is based on a global full-depth ocean and sea-ice configuration of the Massachusetts Institute of Technology general circulation model (MITgcm) and applies an ad-joint approach to generate the physically consistent data assimilations. ECCO2's resolution is high enough to permit the formation of eddies and other narrow current systems within the ocean.

Particles were advected in the surface ocean using TRACMASS (http://tracmass.org), an off-line particle tracking code that calculates trajectories using Eulerian velocity fields. TRACMASS estimates the trajectory path through each grid cell of every Lagrangian particle, using an analytical solution to a differential equation that depends on the velocities on the grid-box walls. The scheme was originally developed for stationary velocity fields^20^,^38^, and thereafter extended for time-dependent fields by solving a linear interpolation of the velocity field both in time and in space over each grid box^39^. This differs from the Runge-Kutta method, where trajectories are iterated forward in time with short time steps.

### Particle seeding and connectivity patches

We seeded six particles in the second depth layer of each ECCO2 grid cell (a total of 4 million particles at each seeding time, or 36 million particles in total over all seeding times). When calculating connectivity, we aggregated the model's 1/4° × 1/4° grid cells to 11,116 discrete 2° × 2° patches. The size of these connectivity patches was selected as a balance of computational feasibility and biogeographic detail. Each connectivity patch is therefore seeded with 384 particles at each seeding event (9 in total). The second depth layer is between 5 and 20 m depth and was used to avoid potential numerical problems due to how ECCO2 implement a varying sea surface, precipitation, and evaporation. See Supplementary Fig. 2 for the spatial distribution of connectivity patches. Particles were seeded at 9 points in time: 1 January 2001, 1 February 2002, 1 March 2003, 1 April 2004, 1 May 2005, 1 June 2006; 1 July 2007, 1 August 2008; and 1 September 2009 in model years. As a consequence of the multiple seeding times, a total of 3,456 particles were used per patch to estimate connectivity. Particles were then advected using horizontal velocity fields from the second depth layer in ECCO2 so that they were locked in the surface ocean. We looped velocity fields for the years 2000–2010 continuously and advected the particles for 100 years in total. Particle positions were saved every 3 days and used to calculate minimum connection times. No extra diffusivity was added to the movement of the particles. Supplementary Figure 6 shows the relationship between advection time and number of other patches reached. It is clear from this figure that the number of connectivity patches reached saturates after about 12 years. In other words, like the number of particles released, there are diminishing returns to running simulations for longer integration times.

### Estimating the timescales of connectivity

The resulting Lagrangian particle trajectories were used to estimate the shortest time taken for water to travel from one patch in the surface ocean to another. This minimum connection time is a variant on the standard measure of ocean distance, which is the expected transit time for water to travel from one patch to another^13^,^14^. We use the minimum and not the expected connection time for two reasons. First, the minimum connection time is a more appropriate metric for phytoplankton and bacterial connectivity since asexually reproducing organisms have high reproductive output that attenuates low dispersal probabilities, and only a few individuals are required to ‘connect' two places, especially in terms of population genetics^22^. It is therefore possible for such organisms to exploit dispersal routes where the probability to reach a given destination is very low. Second, expected transit times in the global ocean are not properly defined, as water can recirculate for an infinitely long time. There is no limit, therefore, to the distribution of connectivity times over which to calculate expected connection times. Thus, the minimum connection time is a preferable alternative measure of ocean distance for this global application.

Minimum connection times for the global surface ocean are called Min-T, and they are stored in the form of a matrix—the Min-T connectivity matrix, where each *i*, *j* element represents the shortest transit time between a given source patch *i* and destination patch *j* (Fig. 1a). The *raw* Min-T matrix, produced from the Lagrangian particle tracking, is highly sparse with most pairs of patches being unconnected.

### Network analysis of shortest/quickest paths

Estimation of connection times between all pairs of patches globally using Lagrangian particle simulations alone would require a currently infeasible number of particles^23^ (see particle density sensitivity test described below). To circumvent this obstacle, a shortest-path algorithm was used to calculate missing values in the raw Min-T connectivity matrix. Here the network is the global ocean, with patches in the ocean as nodes, and minimum connection times as edges connecting the nodes. Applied to this network, shortest path algorithms identify the shortest path between every global ocean patch-pair, accounting for all possible multistep connections (see Supplementary Online Material for details). For example, if there is no direct connection between nodes *A* and *D*, then these algorithms identify the multistep connection from *A*→*B*→*C*→*D*. We use Dijkstra's algorithm^24^, which is one of the most commonly used shortest path algorithms, and which fits our specific application. The end result is a modified Min-T connectivity matrix (Fig. 1b), where all possible minimum connection times between patches are calculated.

Each step along these minimum-time routes may be unlikely, and so the conditional multistep probability (of going from A to B to C to D...) can have a very low probability as well. However, we assume that the effect of these low probabilities is attenuated by the large reproductive output of microorganisms drifting with ocean currents. Over the timescales that we are considering, microorganisms moving with water masses can grow by the million^25^. Hence, there will still be planktonic organisms traveling along the potentially low probability paths identified here. Indeed, if one considers the dispersal of genetic material, then there need only be a small number of individuals traveling along these Min-T routes, to make them evolutionarily relevant^17^,^22^,^25^.

While nodes in a network are usually defined as singular nodes with well-defined distances between them, our ocean patches have relatively large areas and are continuously adjacent to one another. This difference creates a problem when using Dijkstra's algorithm since a particle seeded next to the boundary of its initial patch can rapidly move to an adjacent patch. However, shortest path algorithms assumes that the travel time across each intermediate node is zero, or at least included in the edge distances. (This phenomenon is also a problem when analysing the speed of tracer transport in General Circulation Models^40^.) By removing all calculated connectivity times shorter than 1 year before applying the shortest-path algorithm, we limit the effect of not including within-patch crossing times. The 365-day cutoff is based on calculated typical residence times in the patches, which are on the order of weeks. All initial minimum connectivity times are based on travel distances at least an order of magnitude longer than typical patch crossing distances.

The removed connectivity times were added back to the final connectivity matrix, allowing for connection times shorter than 365 days, as shown in Figs 2 and 3. It should be noted that the absolute number of connectivity times shorter than 1 year in Fig. 3 are identical for the Raw and Dijkstra cases. The lack of discontinuities between sub-annual and longer connection times in Fig. 2 (and all other cases we have explored) give us confidence that the resulting connectivity matrix is reasonable and that our approach works.

After applying Dijkstra's algorithm, we find that the resulting minimum connection time matrices are all connected. However, we do find some areas that are only connected in one direction (that is, there are connection time to, but not from, particular regions). These areas are mainly inland seas—the Baltic and Mediterranean, for example. However, they only account for a small fraction (2%) of the modified Min-T matrix and, consequently, do not impact the general result of the timescales of global surface ocean connectivity.

### Particle seeding sensitivity test

Since the number of particles seeded per grid-cell and the seeding times are limited, we have not accounted for all possible Min-T pathways. As a result, our estimates of the timescales of global surface connectivity are conservative, since adding more particles and seeding dates could only lead to shorter Min-T pathways (that is, we look for the shortest connection times over all possibilities including seeding times). Thus, the few seeding dates—although arguably numerically incomplete—strengthen our conclusion that the global surface ocean is well connected over a few decades.

To examine the effect of particle seeding density, we performed a particle sensitivity test. Minimum connection times from a patch in the north pacific to all others were estimatedusing simulations with increasing numbers of seeded particles. Supplementary Figure 3 shows the results of these simulations. It is clear that a larger oceanic extent is reached as the number of particles released increases. However, when we examine only those patches that were reached in all seeding experiments (Supplementary Fig. 4), we can see that increasing the number of particles serves only to decrease the minimum connection times in these patches (Supplementary Fig. 4: with 84 particles some areas are reached after 100 years—the patches in gold, in contrast with 16,660 particles, these same patches are reached after around 20 years—patches now in light red).

Finally, we show the aggregated results of the sensitivity test in Supplementary Fig. 5, where we plot the fraction of patches reached (over the whole ocean) and the median minimum connection time from this study patch. The fraction of patches reached saturates at around 30%, which means that there are diminishing returns (in terms of estimating minimum connection times to new patches) to adding more particles. It also indicates that, to release enough particles to estimate minimum connection times to all patches globally, a currently impossible number of Lagrangian particles would be required. Similarly, the median minimum connection time from this patch saturates at around 8,000 particles released. In our simulations we use 3,456 particles per patch as this achieved a balance of connectivity sampling power and computational efficiency.

## Additional information

**How to cite this article:** Jönsson, B.F. & Watson, J.R. The timescales of global surface-ocean connectivity. *Nat. Commun.* 7:11239 doi: 10.1038/ncomms11239 (2016).

## Supplementary Material

Supplementary Information Supplementary Figures 1-6

## Figures and Tables

**Table 1:** Median minimum connectivity time.

Destination					
Source	Arctic	Atlantic	Indian	Pacific	Southern
Arctic	3.7 (1.9)	5.8 (2.6)	8.6 (2.0)	8.8 (1.9)	11.2 (1.6)
Atlantic	6.4 (2.2)	3.5 (2.3)	7.7 (2.6)	9.2 (1.8)	7.9 (3.0)
Indian	8.3 (2.1)	5.5 (1.9)	2.3 (3.1)	5.2 (2.0)	5.0 (2.5)
Pacific	8.1 (2.3)	7.2 (1.7)	3.9 (2.1)	3.4 (1.7)	5.9 (1.7)
Southern	9.1 (2.0)	6.4 (2.2)	5.0 (2.2)	6.2 (1.8)	4.1 (1.8)

Median minimum connectivity time between ocean basins in years. S.d. in parentheses.

## References

[b1] Baas-Becking L. Geobiologie of Inleiding tot de Milieukunde ed Van Stockum W. P., Zoon The Hague (1934).

[b2] Fenchel T. & Finlay B. J. The ubiquity of small species: patterns of local and global diversity. Bioscience 54, 777–784 (2004).

[b3] De Wit R. & Bouvier T. 'Everything is everywhere, but, the environment selects'; what did Baas Becking and Beijerinck really say? Environ. Microbiol. 8, 755–758 (2006). 16584487 10.1111/j.1462-2920.2006.01017.x

[b4] McGillicuddy D. J. . Eddy/wind interactions stimulate extraordinary mid-ocean plankton blooms. Science 316, 1021–1026 (2007). 17510363 10.1126/science.1136256

[b5] Thomas M. K., Kremer C. T., Klausmeier C. A. & Litchman E. A global pattern of thermal adaptation in marine phytoplankton. Science 338, 1085–1088 (2012). 23112294 10.1126/science.1224836

[b6] Martiny J. . Microbial biogeography: putting microorganisms on the map. Nat. Rev. Microbiol. 4, 102–112 (2006). 16415926 10.1038/nrmicro1341

[b7] Casteleyn G. . Limits to gene flow in a cosmopolitan marine planktonic diatom. Proc. Natl Acad. Sci. USA 107, 12952–12957 (2010). 20615950 10.1073/pnas.1001380107 PMC2919969

[b8] Saez A. G. . Pseudo-cryptic speciation in coccolithophores. Proc. Natl Acad. Sci. USA 100, 7163–7168 (2003). 12759476 10.1073/pnas.1132069100 PMC165847

[b9] Rynearson T. A. & Virginia Armbrust E. Genetic differentiation among populations of the planktonic marine diatom Ditylum brightwellii (Bacillariophyceae). J. Phycol. 40, 34–43 (2004).

[b10] Sul W. J., Oliver T. A., Ducklow H. W., Amaral-Zettler L. A. & Sogin M. L. Marine bacteria exhibit a bipolar distribution. Proc. Natl Acad. Sci. USA 110, 2342–2347 (2013). 23324742 10.1073/pnas.1212424110 PMC3568360

[b11] Godhe A. . Seascape analysis reveals regional gene flow patterns among populations of a marine planktonic diatom. Proc. R. Soc. B Biol. Sci. 280, 1773 (2013). 10.1098/rspb.2013.1599 PMC3826216 24174105

[b12] Froyland G., Stuart R. M. & van Sebille E. How well-connected is the surface of the global ocean? Chaos 24, 3 (2014). 10.1063/1.4892530 25273206

[b13] Alberton F. . Isolation by oceanographic distance explains genetic structure for Macrocystis pyrifera in the Santa Barbara Channel. Mol. Ecol. 20, 2543–2554 (2011). 21535280 10.1111/j.1365-294X.2011.05117.x

[b14] Watson J. R. . Currents connecting communities: nearshore community similarity and ocean circulation. Ecology 92, 1193–1200 (2011). 21797147 10.1890/10-1436.1

[b15] Mitarai S., Siegel D. & Winters K. A numerical study of stochastic larval settlement in the California Current system. J. Mar. Syst. 69, 295–309 (2008).

[b16] Cowen R., Paris C. & Srinivasan A. Scaling of connectivity in marine populations. Science 311, 522–527 (2006). 16357224 10.1126/science.1122039

[b17] Kool J. T., Paris C. B. & Andre S. Complex migration and the development of genetic structure in subdivided populations: an example from Caribbean coral reef ecosystems. Evolution, September 2009, 1–10 (2010).

[b18] Mora C. . High connectivity among habitats precludes the relationship between dispersal and range size in tropical reef fishes. Ecography 35, 89–96 (2012).

[b19] Wood S., Paris C. B., Ridgwell A. & Hendy E. J. Modelling dispersal and connectivity of broadcast spawning corals at the global scale. Global Ecol. Biogeogr. 23, 1–11 (2014).

[b20] Döös K. Interocean exchange of water masses. J. Geophys. Res. 100, 13499–13514 (1995).

[b21] Cowen R., Gawarkiewicz G., Pineda J., Thorrold S. & Werner F. E. Population connectivity in marine systems. Oceanography 20, 14–20 (2007).

[b22] Hedgecock D., Barber P. H. & Edmands S. Genetic approaches to measuring connectivity. Oceanography 20, 70–79 (2007).

[b23] Simons R. D., Siegel D. A. & Brown K. S. Model sensitivity and robustness in the estimation of larval transport: A study of particle tracking parameters. J. Mar. Syst. 119-120, 19–29 (2013).

[b24] Dijkstra E. W. A note on two problems in connexion with graphs. Numerische Mathematik 1, 269–271 (1959).

[b25] Falkowski P. G. . The evolution of modern eukaryotic phytoplankton. Science 305, 354–360 (2004). 15256663 10.1126/science.1095964

[b26] Wunsch C., Heimbach P., Ponte R. M. & Fukumori I. The ECCO-GODAE Consortium Members. The global general circulation of the ocean estimated by the ECCO-consortium. Oceanography 22, 88–103 (2009).

[b27] Ebbesmeyer C. C. & Ingraham W. J. Shoe spill in the North Pacific. Eos Trans. Am. Geophys. Union 73, 361–365 (1992).

[b28] Ebbesmeyer C. C. & Ingraham W. J. Pacific toy spill fuels ocean current pathways research. Eos Trans. Am. Geophys. Union 75, 425–430 (1994).

[b29] Schaum E., Rost B., Millar A. J. & Collins S. Variation in plastic responses of a globally distributed picoplankton species to ocean acidification. Nat. Clim. Change 3, 298–302 (2012).

[b30] Barton A. D., Dutkiewicz S., Flierl G., Bragg J. & Follows M. J. Patterns of diversity in marine phytoplankton. Science 327, 1509–1511 (2010). 20185684 10.1126/science.1184961

[b31] Jacobi M. N., André C., Döös K. & Jonsson P. R. Identification of subpopulations from connectivity matrices. Ecography 35, 1004–1016 (2012).

[b32] Jacobi M. N. & Jonsson P. R. Optimal networks of nature reserves can be found through eigenvalue perturbation theory of the connectivity matrix. Ecol. Appl. 21, 1861–1870 (2011). 21830724 10.1890/10-0915.1

[b33] Watson J. R. . Identifying critical regions in small-world marine metapopulations. Proc. Natl Acad. Sci. USA 108, 907–913 (2011). 10.1073/pnas.1111461108 PMC3203776 21987813

[b34] Treml E., Halpin P., Urban D. & Pratson L. Modeling population connectivity by ocean currents, a graph-theoretic approach for marine conservation. Landscape Ecol. 23, 19–36 (2008).

[b35] Chust G., Irigoien X., Chave J. & Harris R. P. Latitudinal phytoplankton distribution and the neutral theory of biodiversity. Global Ecol. Biogeogr. 22, 531–543 (2013).

[b36] Sarmiento J. L. . Response of ocean ecosystems to climate warming. Global. Biogeochem. Cycles. 18, GB3003 (2004).

[b37] Treml E. A. & Halpin P. N. Marine population connectivity identifies ecological neighbors for conservation planning in the Coral Triangle. Conserv. Lett. 5, 441–449 (2012).

[b38] Blanke B. & Raynaud S. Kinematics of the pacific equatorial undercurrent: An Eulerian and Lagrangian approach from GCM results. J. Phys. Oceanogr. 27, 1038–1053 (1997).

[b39] de Vries P. & Döös K. Calculating Lagrangian trajectories using time-dependent velocity fields. J. Atmos. Oceanic Technol. 18, 1092–1101 (2001).

[b40] Griffies S. M. Elements of the Modular Ocean Model (MOM): 2012 release. Geophysical Fluid Dynamics Laboratory (GFDL) Ocean Group Technical Report No. 7, 1–631 (2012).

