# Supplementary material for: The timescales of global surface-ocean connectivity
Source: Nat Commun. 2016 Apr 19;7:11239. doi: 10.1038/ncomms11239 (PMC4838858; doi:10.1038/ncomms11239)
Supplement: Supplementary Information — Supplementary Figures 1-6 [file ncomms11239-s1.pdf]

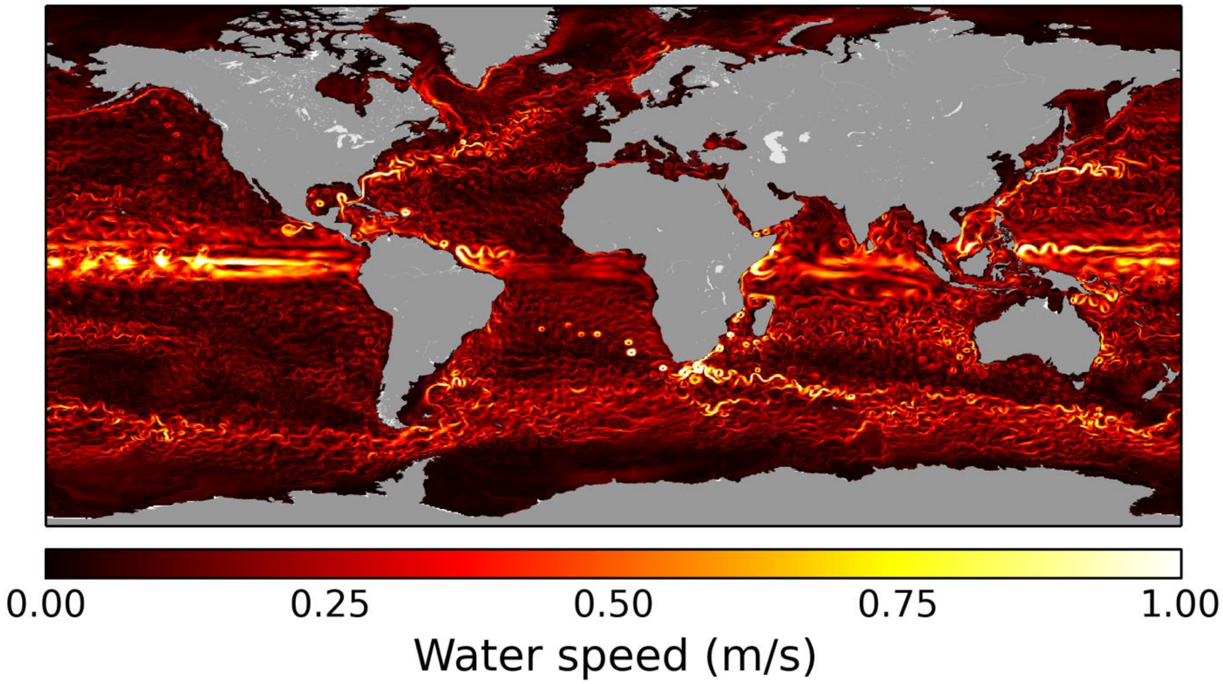

Supplementary Fig. 1: ECCO2 Velocities Jan 1st 2000
A snapshot of the ECCO2 surface velocity field (m/s) from January 1st, 2000. The model has global coverage, and a ¼° horizontal resolution. Eddies and other meso-scale features that affect dispersal in the ocean are represented in the model.

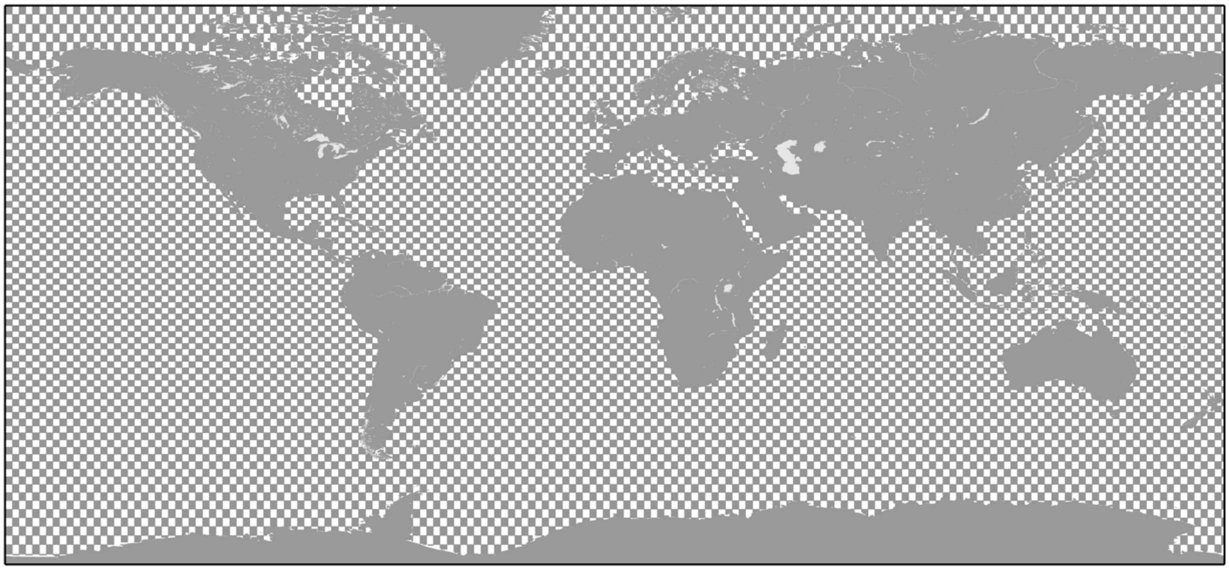

Supplementary Fig. 2: Connectivity patches
The white and grey checkerboard over ocean areas identifies the patches used to calculate surface connectivity. Each patch is approximately 2° × 2°.

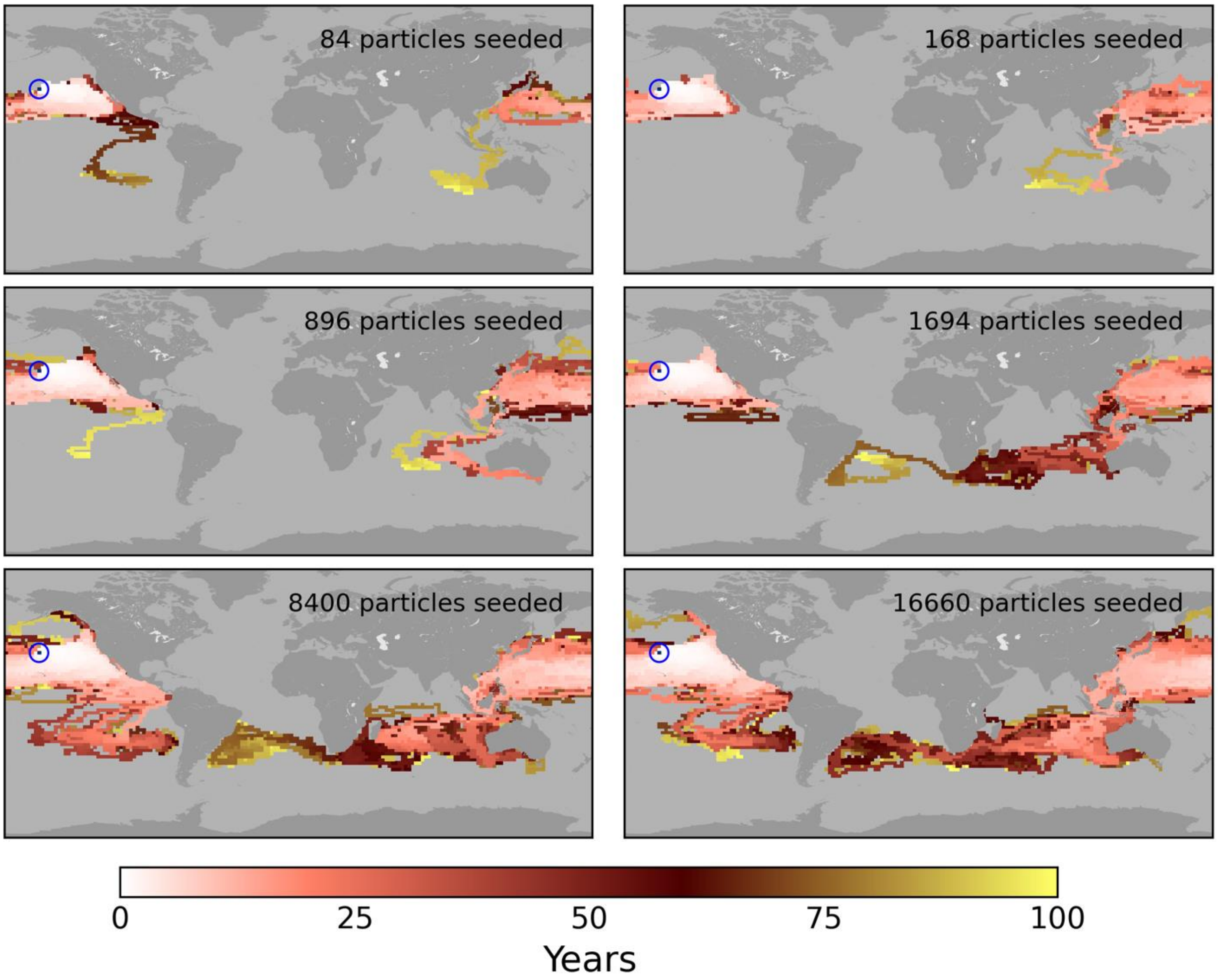

Supplementary Fig. 3: Effect of Seeding density on distance
Sensitivity test for the number of seeded particles. Minimum connection times from a source patch in the north Pacific are estimated using simulations seeded with different numbers of Lagrangian particles.

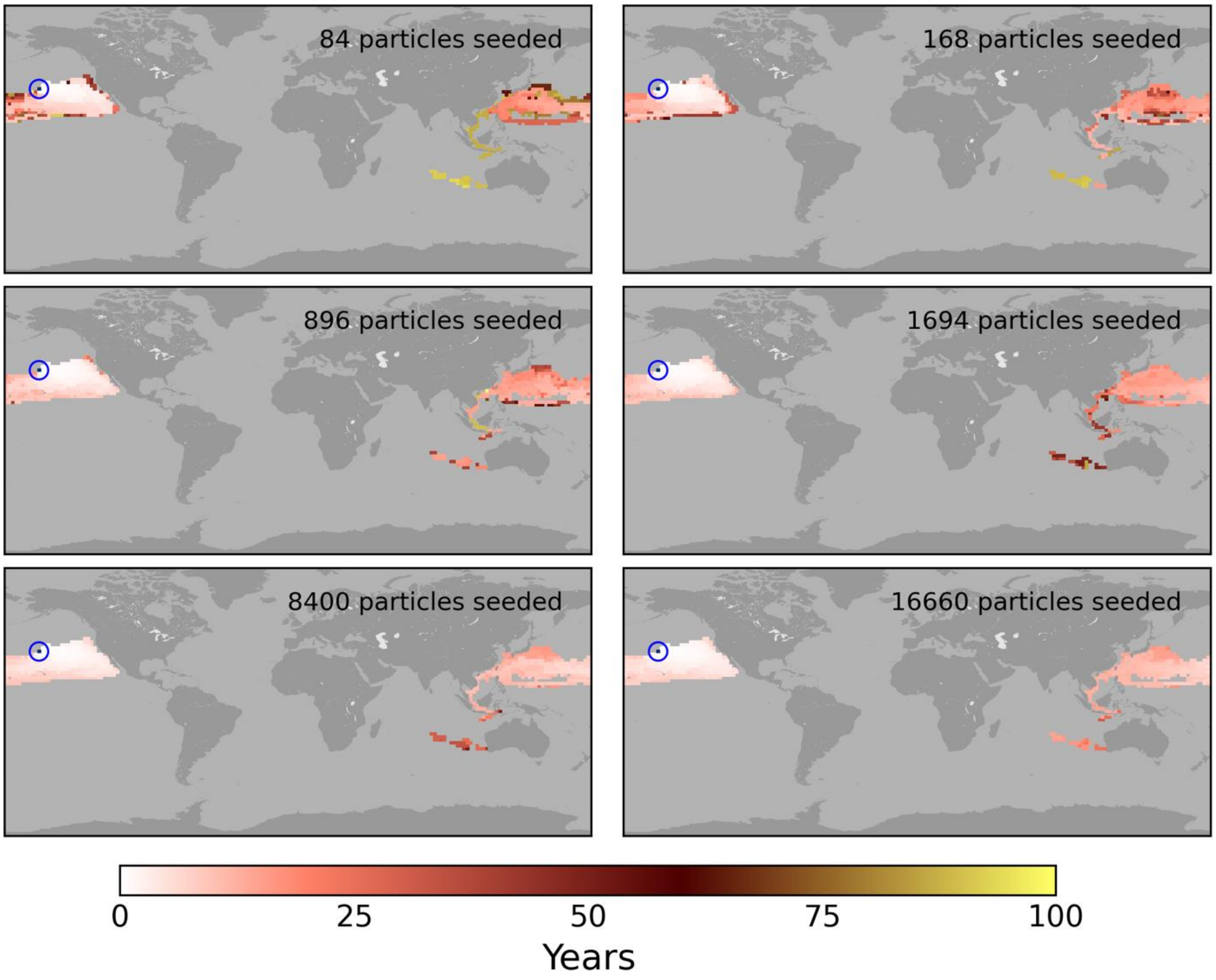

Supplementary Fig. 4: Effect of Seeding density on time
Sensitivity test for the number of seeded particles. Minimum connection times from a source patch in the north Pacific are estimated using simulations with a range of Lagrangian particles. Here, only patches reached in **all** particle seeding simulations are shown (hence the contraction in range from Supplementary Fig. 3).

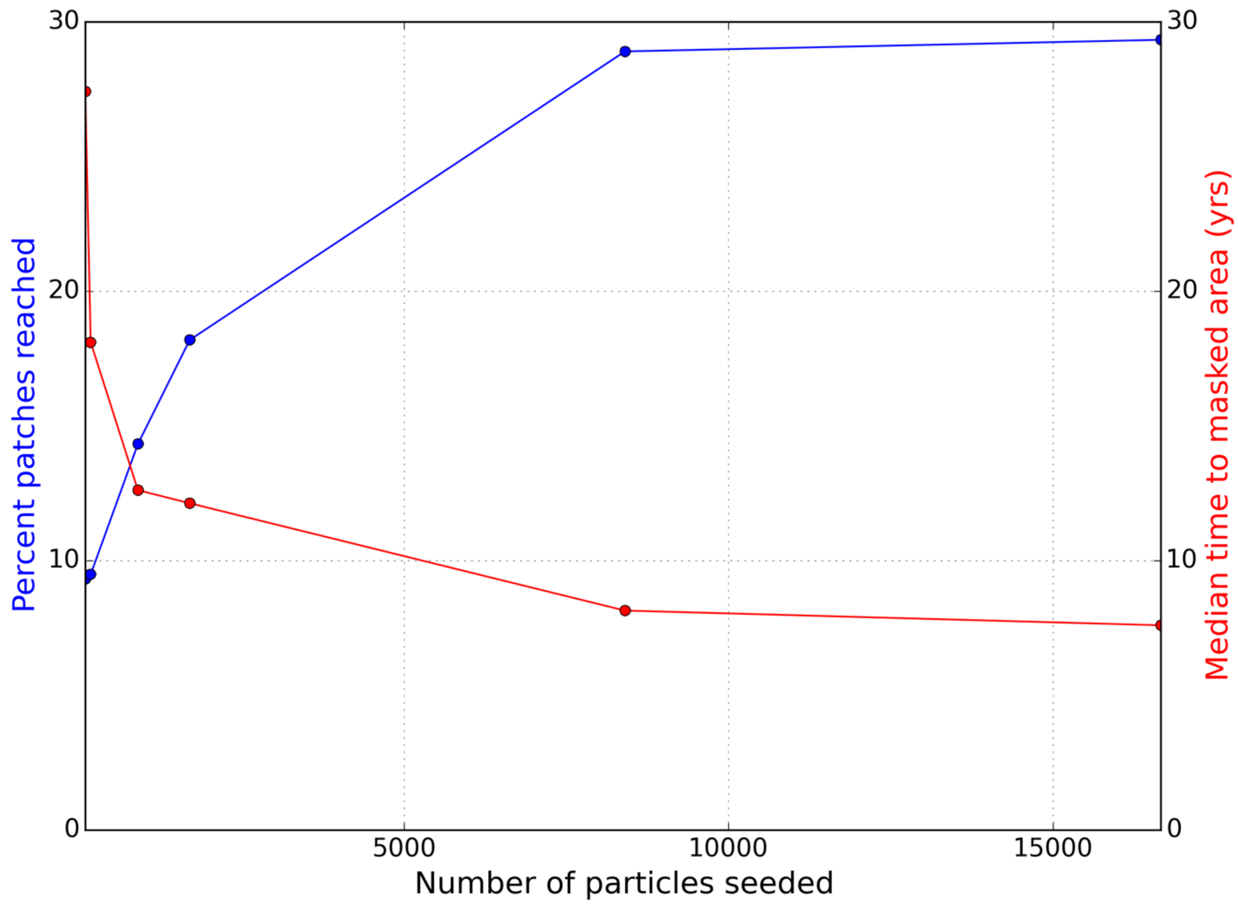

Supplementary Fig. 5: Seeding density vs time and patches reached
The fraction of total number connectivity patches, over the global ocean, reached as a function of number of particles released (blue line) and the median minimum connection time from the source patch in the north Atlantic to the ``masked patches" -- those patches reached in all particle seeding simulations (i.e., corresponding to those in Supplementary Fig. 4).

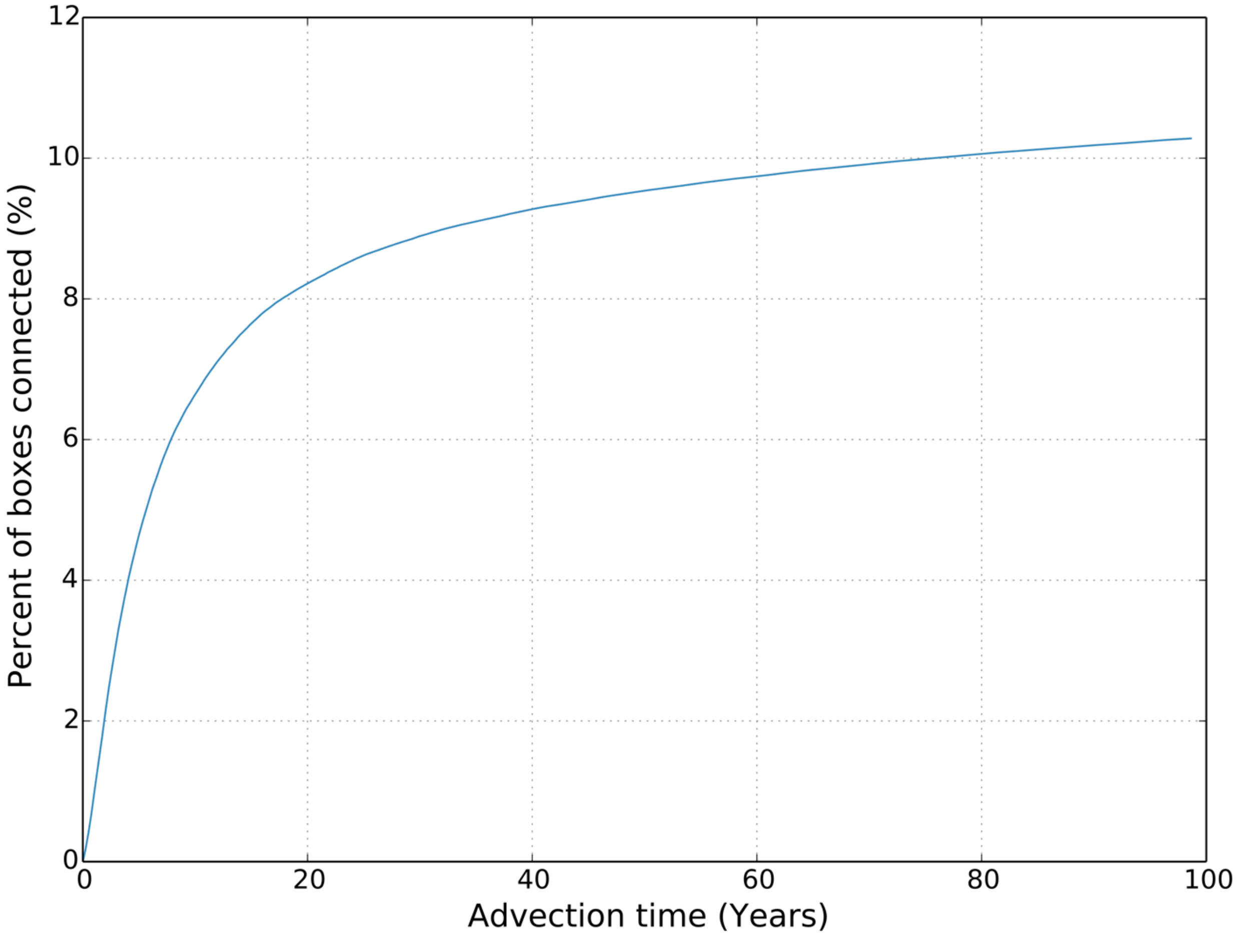

Supplementary Fig. 6: Connected patches vs time
Fraction of the total number of patches connected as a function of advection time. The relationship is saturating, indicating marginal gains by running the Lagrangian particle tracking for longer integration periods.
